# Predicting drug characteristics using biomedical text embedding

**DOI:** 10.1186/s12859-022-05083-1

**Published:** 2022-12-07

**Authors:** Guy Shtar, Asnat Greenstein-Messica, Eyal Mazuz, Lior Rokach, Bracha Shapira

**Affiliations:** Department of Software and Information Systems Engineering, Ben-Gurion University of the Negev, Beer-Sheva, Israel

**Keywords:** Drug interactions, Text mining, Machine learning

## Abstract

**Background:**

Drug–drug interactions (DDIs) are preventable causes of medical injuries and often result in doctor and emergency room visits. Previous research demonstrates the effectiveness of using matrix completion approaches based on known drug interactions to predict unknown Drug–drug interactions. However, in the case of a new drug, where there is limited or no knowledge regarding the drug’s existing interactions, such an approach is unsuitable, and other drug’s preferences can be used to accurately predict new Drug–drug interactions.

**Methods:**

We propose adjacency biomedical text embedding (ABTE) to address this limitation by using a hybrid approach which combines known drugs’ interactions and the drug’s biomedical text embeddings to predict the DDIs of both new and well known drugs.

**Results:**

Our evaluation demonstrates the superiority of this approach compared to recently published DDI prediction models and matrix factorization-based approaches. Furthermore, we compared the use of different text embedding methods in ABTE, and found that the concept embedding approach, which involves biomedical information in the embedding process, provides the highest performance for this task. Additionally, we demonstrate the effectiveness of leveraging biomedical text embedding for additional drugs’ biomedical prediction task by presenting text embedding’s contribution to a multi-modal pregnancy drug safety classification.

**Conclusion:**

Text and concept embeddings created by analyzing a domain-specific large-scale biomedical corpora can be used for predicting drug-related properties such as Drug–drug interactions and drug safety prediction. Prediction models based on the embeddings resulted in comparable results to hand-crafted features, however text embeddings do not require manual categorization or data collection and rely solely on the published literature.

## Introduction

Drug–drug interactions (DDIs) are a leading cause of death, as well as high healthcare costs [1]. The number of patients injured by drug interactions is estimated to be between 3 and 5% of all hospital medication errors. Drug interactions also account for a large number of patient visits to doctors and emergency rooms [2, 3]. Thirty-six percent of older Americans use five or more drugs or supplements on a daily basis, and 15% are at risk of a significant Drug–drug interaction (DDI) [4]. Potential DDIs are usually discovered after the third phase of a clinical trial or even after a medication has been approved. The issue of DDIs is not explicitly addressed in the clinical trials for new medicines. The most feasible approach for investigating the vast number of drug combinations to screen interacting medicines is in-silico Drug–drug interaction detection.

DDIs and adverse drug reactions (ADRs) can be investigated using a population-based approach in in-populo pharmacoepidemiology research. For example, in a series of case-control and case-crossover population-based studies utilizing US Medicaid data, the interactions between warfarin and various antibiotics were assessed for increased risk of gastrointestinal bleeding and hospitalization [5].

Computational methods have gained popularity for DDI prediction, saving time and money [6]. These methods fall into three categories: literature-based extraction methods, machine learning-based prediction methods, and pharmacovigilance-based data mining methods. Literature-based extraction methods detect DDIs from published literature using natural language processing (NLP) techniques; machine learning-based prediction methods build prediction models based on the known DDIs in databases and predict novel ones [7–9]; pharmacovigilance-based data mining methods usually apply statistical techniques on various electronic data to detect Drug–drug interaction signals. However, machine learning methods that combine the structured data of known databases with unstructured textual information publicly available have not yet been proposed.

Distributed vector representations or embeddings, which map variable length text to dense fixed length vectors and capture prior knowledge which can be transferred to downstream tasks, have become the de facto standard for text representation in deep learning-based NLP tasks in both clinical and more general domains.

In this paper, we evaluate the contribution of combining different biomedical text embedding methods with a machine learning model based on matrix factorization, which, although reported high performances, is limited in cases where few or no interactions are available for the examined drug. Our contributions are as follows:

1. We demonstrate the effectiveness of combining structural data and biomedical text embeddings for DDI prediction and pregnancy drug safety classification.
2. We compare different biomedical text embedding methods and show that the concept embedding approach, which involves biomedical information in the embedding process, is superior for both DDI prediction and pregnancy drug safety classification.
3. We introduce adjacency biomedical text embedding (ABTE), a hybrid approach which combines known drugs’ interactions and drugs’ biomedical text embeddings to predict DDIs for both new and well-known drugs. We demonstrate the superiority of this approach compared to adjacency matrix factorization with propagation (AMFP) [8], that represents a state of the art algorithm which is based on matrix factorization.

Where applicable, the methods are evaluated using retrospective analysis, and compared to existing state-of-the-art methods.

## Materials and methods

### Problem formulation

Given *n* drugs, we use $D=d_1,d_2,..,d_n$ to denote the set of drugs, and $R \in R_{n\times n}$ is the interaction association matrix. $r_{ij}=1$ denotes an interaction between drug $d_i$ and drug $d_j$, otherwise $r_{ij}=0$. However, $r_{ij}=0$ does not necessarily mean that there are no interactions between drugs *i* and *j*; it could be a case in which a interaction has not yet been discovered. We use two versions of the interaction association matrix *R*. For two points in time $t < t'$, *R* denotes the association matrix constructed using known interactions at time *t*, and $R'$ denotes the association matrix using the known interactions at time $t'$.

In addition, each drug $d_i$ is represented by a Bio-Text embedding vector $L_i \in R_e$. The Bio-Text embedding vector is generated using state-of-the-art language model on a biomedical domain corpora [10, 11]. Here, *e* is the embedding dimension.

In the Drug–drug interaction prediction problem, we provide the association matrix *R* and the drugs’ name embedding matrix *L* as the training data set to the algorithm. Our goal is to predict the list of interactions that are not present in *R* but appear in the association matrix $R'$. The training release date is referred to as *t*, and the test release date is referred to as $t'$.

### Adjacency biomedical text embedding (ABTE) approach

The ABTE approach combines the advantages of matrix factorization-based methods, where drugs in the interaction space are projected into a low-dimensional space for potential DDI prediction, and a deep learning based classifier, which leverages drugs’ text embeddings derived using state-of-the-art algorithms applied on a biomedical corpora. The ABTE approach is composed of three components: the biomedical text (bio-text) component, the adjacency matrix factorization with propagation (AMFP) component, and a hybrid component which uses a neural network classifier as a stacking model to combine the predictions of the bio-text and AMFP components. Since a matrix factorization-based approach is ineffective in cases where there are limited or no known drug interactions (mainly in the case of a new drug), the ABTE hybrid approach uses a switching criterion to select either the bio-text component or the stacking component, when predicting if there will be an interaction between two drugs *i* and *j*. In case the number of interactions in the training dataset for one of the drugs is lower than *M*, the AMFP latent representation, which is based on the drug’s known interactions is not informative and the bio-text component is used for the interaction prediction. When the number of interactions of both drugs is equal or higher than *M*, the AMFP latent representation is informative and the stacking component is used for the interaction prediction.

1 $$\begin{aligned} \hat{r}_{ij}= {\left\{ \begin{array}{ll} \hat{r}_{ij}^{Stacking}, &{} \text{ if } I_{i} ,I_{j}\ge M\\ \hat{r}_{ij}^{Text}, &{} \text {otherwise} \end{array}\right. } \end{aligned}$$

**Fig. 1:**
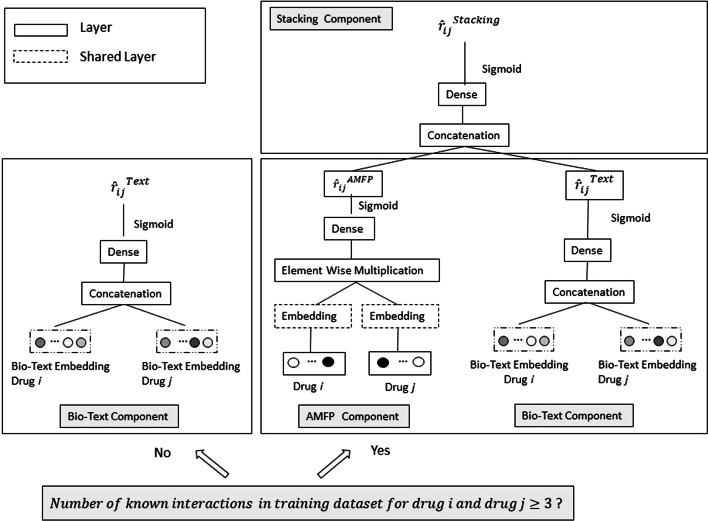
Adjacency Biomedical Text Embedding (ABTE) approach. A hybrid model for Drug–drug interaction prediction, with a switching criterion between prediction based on the Bio-Text only component and a stacking model which combines the predictions of the bio-text component with those of the adjacency matrix factorization (AMFP) [8] component. The switching criterion is based on the number of drugs’ interactions in the training dataset. For drugs with small number of interactions the Bio-Text only component is used, and the stacking model is used otherwise. The input to the Bio-Text component includes the drugs’ pre-trained bio-text embedding, whereas the input to the AMFP component includes the drugs’ id encoded as one hot vector, serving as input to an embedding layer. The embedding layer is shared among the two input drugs, and it is learnt as part of the overall learning architecture. The output of the Bio-Text and the AMFP components is the Drug–drug interaction score between the two input drugs. It is used as input to the stacking model, which provides the final Drug–drug interaction prediction for the hybrid model

Here, ${\hat{r}_{ij}}$ refers to the predicted Drug–drug interaction between drugs *i* and *j*. $\hat{r}_{ij}^{Text}$ is the biomedical text component’s prediction, and $\hat{r}_{i,j}^{Stacking}$ is the stacking component’s prediction. $I_i$, $I_j$ refer to the number of interactions drugs *i* and *j* have in the training set respectively. We used a validation data set to set the value of *M*. For our use case, *M* equals 3 provides the highest model classification accuracy. Figure 1 presents a schematic description of the ABTE approach, and each component is described below.

#### Bio-text component

In the bio-text component, the pre-trained text embedding vectors of the input drugs are fed directly to the first hidden layer of a neural network. We denote the output of the text embedding layer $a^{(0)}$ for a pair of drugs *i*, *j* as the concatenated vector of the two bio-text embedding vectors $L_i$, $L_j$:

2 $$\begin{aligned} a^{(0)}=[L_i,L_j] \end{aligned}$$

Then *a*(0) is fed to the deep neural network, and the forward process is:

3 $$\begin{aligned} a^{(l+1)}= \rho (W^{(l)}a^{(l)}+b^{(l)}) \end{aligned}$$

where *l* is the layer’s depth, and $\rho$ is the ReLU activation function. *a*(*l*), *W*(*l*), and *b*(*l*) are respectively the output, model weight, and bias of layer *l*. The last layer is fed to a sigmoid function for DDI prediction based on the drugs’ text embeddings $\hat{R}^{Text}$. *H* is the number of hidden layers.

4 $$\begin{aligned} \hat{R}^{Text} = \sigma (W^{H+1}a^H+b^{H+1}) \end{aligned}$$

Since DDI interaction prediction is a classification problem, we used the binary cross-entropy loss function *L* to train the model.

5 $$\begin{aligned} L= \Sigma _{i,j\epsilon Y} R_{ij}log(\hat{R}_{ij}^{Text})-(1-R_{ij})log(1-\hat{R}_{ij}^{Text}) \end{aligned}$$

Here, *Y* is the set of instances (drug pairs), $R_{ij}$ are the true labels which represent the existence or absence of a drug interaction, and $\hat{R}_{ij}^{Text}$ is the predicted value. *Y* is created using negative sampling, where all positive samples (existing drug interactions at time *t*) are used, and a sample of the negative instances is selected randomly in each training epoch. The sample ratio is a model hyperparameter that should be tuned using a validation set (see Implementation section). We used one negative instance for each positive instance during training. A different sample was selected in each iteration. The network weights $W^{(l)}$ and biases $b^{(l)}$ are learned during the training process. A negative, non-existing interaction might represent an undiscovered but existing interaction [12, 13]. Undiscovered interactions share common characteristics with negative samples in recommender systems, which result from implicit feedback from the user. Negative sampling is a common practice in training models from implicit feedback [14] and interaction data [15, 16].

#### Adjacency matrix factorization with propagation (AMFP) component

The adjacency matrix factorizaiton (AMF) component [8] applies matrix factorization on the adjacency matrix of Drug–drug interactions and shares the latent factors of each drug between rows and columns. Then the AMF model exploits adaptive moment estimation (Adam) optimization to optimize the weights of the element-wise multiplication and the biases. Adjacency matrix factorization with propagation (AMFP) is an extension of AMF that adds a step in which the latent factors of each drug are propagated to the interacting drugs. The latent factor propagation is controlled by a propagation factor that controls the information passed from the neighborhood of interacting drugs. The latent factor of each node is shared with the node’s neighborhood. When the value of the propagation factor reaches zero, AMF is equivalent to AMFP.

#### Stacking component

To combine the AMFP model’s prediction with the bio-text model’s prediciton, a stacking model is used. We train each model separately and then feed each model’s prediction output for each instance to a multilayer neural network, with a sigmoid activation function in the last layer and the binary cross-entropy loss function.

### Bio-text embedding models

We evaluate the following bio-text embedding models:

- *BioConceptVec concept embedding* BioConcpetVec, proposed by [11], is a state-of-the-art biomedical concept text embedding model. It is trained by implying PubTator’s state-of-the-art name entity recognition (NER) tool to identify and normalize medical concepts [17], and then four machine learning models (Word2Vec CBOW, Word2Vec Skip-gram, GloVe, and FastText) are trained to calculate text and concept embeddings based on  30 million PubMed abstracts. To match the drug names to the BioConceptVec concepts, we leverage PubTator’s central chemical mapping. We evaluate different machine learning models, which were used to train the BioConceptVec embeddings. We present the results of the Word2Vec CBOW embedding flavor as part of the evaluation, as it provided superior or comparable results to the other embedding flavors.
- *BioConceptVec text embedding* Although BioConceptVec includes more than 400,000 medical concepts, we were only able to directly match about 70% of the drugs in our dataset to BioConceptVec concepts. Therefore, we evaluate the effectiveness of using either concept embedding or text embedding, where the drug name is explicitly used to match the vocabulary of the BioConceptVec embeddings. We present the results of the Word2Vec CBOW embedding flavor as part of the evaluation, as it provided superior or comparable results to the other embedding flavors.
- *BioBERT text embedding* BioBERT, proposed by [18], is a state-of-the-art domain-specific language representation model pretrained on large-scale biomedical corpora (PubMed abstracts and PubMed Central full-text articles). The BioBERT model is based on BERT, a contextualized word representation language model that obtains state-of-the-art performance on most NLP tasks. BioBERT has been shown to outperform previous state-of-the-art language models on different representative biomedical text mining tasks ([18, 19]), such as biomedical NER, biomedical relation extraction from text, and biomedical question answering.

### Implementation

We implemented the different flavors of the ABTE approach using Keras. We used a public Python implementation and evaluation methods for the AMFP baseline.

To determine the hyperparameters of the ABTE approach, we use 20% of the training set as a validation set. All weights were randomly initialized using the Glorot normal initializer. The following batch sizes were used: 128, 256, 512, and 1,024, and learning rates 0.1, 0.01, 0.001 and 0.0001 were tested. We evaluated the following number of factors (embedding sizes): 32, 64,128, 256, 512, and 1,024 for the drug-ID embedding, as well as dropout levels in the range of 0-0.9 in steps of 0.1, the number of epochs in the range of 1–50 with steps of 5, and propagation factors in the range of 0.0–1.0 in steps of 0.1. The ABTE switching criterion value 3 was optimized using each value in the range of 0–10. The ratio of negative to positive samples was tuned using each value in the range of 1-10. We used the text embedding size of the pre-trained models, which is 100.

## Results

### Drug–drug interaction prediction

**Table 1:** DDI prediction-ABTE approach

Approach	Concept		Word		BERT	
	AUC	AUPR	AUC	AUPR	AUC	AUPR
Text Concate	0.78	0.36	0.74	0.32	0.74	0.30
Text Multiply	0.75	0.34	0.73	0.31	0.73	0.28
Text Concate, Feature Combination	0.75	0.32	0.73	0.30	0.72	0.27
Text Multiply, Feature Combination	0.74	0.31	0.72	0.28	0.71	0.26
Text Concate, Stacking	**0.81**	**0.48**	0.78	0.47	0.73	0.43
Text Multiply, Stacking	0.80	0.47	0.79	0.46	0.80	0.46

Best scores are highlighted in bold

Comparison of different Bio-Text embedding models and ABTE flavors to combine Bio-Text embedding and known interactions

**Table 2:** DDI prediction for rare drugs (less than three known interactions in the training data set)-ABTE approach

Approach	Concept		Word		BERT	
	AUC	AUPR	AUC	AUPR	AUC	AUPR
Text Concate	**0.76**	**0.27**	0.69	0.22	0.70	0.23
Text Multiply	0.69	0.22	0.69	0.23	0.70	0.21
Text Concate, Feature Combination	0.70	0.22	0.68	0.20	0.68	0.19
Text Multiply, Feature Combination	0.68	0.20	0.67	0.20	0.66	0.17
Text Concate, Stacking	0.65	0.22	0.57	0.19	0.43	0.13
Text Multiply, Stacking	0.66	0.22	0.55	0.19	0.70	0.19

Best scores are highlighted in bold

Comparison of different Bio-Text embedding models and ABTE flavors to combine text embedding and known interactions

**Table 3:** DDI prediction-Matrix Factorization based models

Approach	Full		Rare	
	AUC	AUPR	AUC	AUPR
AMF	0.76	0.35	0.62	0.17
AMFP	0.79	0.46	0.63	0.21
SSI DDI	0.63	0.24	0.60	0.16
ISCMF	0.74	0.36	0.68	0.23
DeepFM	0.75	0.34	0.59	0.17
Text Concate	0.78	0.36	**0.76**	**0.27**
Text Concate, Stacking	**0.81**	**0.48**	0.65	0.22

Best scores are highlighted in bold

Comparison between the ABTE best flavors and Matrix Factorization based models. The comparison covers state of the art models for DDI, as well as hybrid approaches to combine text embedding and known interactions. These models support both small molecule and biological drugs. The table compares the entire test data set as well as rare drugs only (drugs with less than three known interactions in the training data set)

**Table 4:** DDI prediction for small molecule drugs

Approach	Full		Rare	
	AUC	AUPR	AUC	AUPR
AMFP	0.79	0.47	0.61	0.21
Chemprop	0.70	0.31	0.63	0.21
CASTER	0.73	0.32	0.69	0.24
SSI DDI	0.61	0.24	0.58	0.16
ISCMF	0.75	0.38	0.69	0.24
Text Concate	0.77	0.38	**0.75**	**0.28**
Text Concate, Stacking	**0.80**	**0.48**	0.66	0.25

Best scores are highlighted in bold

Comparison between the ABTE best flavors to state of the art DDI prediction models, as well as to hybrid approaches which combine text embedding and known interactions. These comparison includes models which are based on the chemical structure of the drug, and are limited to small molecule drugs only. The table compares the entire test set as well as rare drugs only (drugs with less than three known interactions in the training data set)

In this section, we present our experiments, which are aimed at evaluating the contribution of adding text embedding information to DDI prediction in general, and specifically for predicting interactions for new drugs where there is limited or no knowledge regarding existing interactions. As a baseline, we use the AMFP model, which is considered the state-of-the-art benchmark ([8]). Our evaluation includes a comparison of different variants of the ABTE approach, as well as different pretrained bio-text embedding of the drug names. Our evaluation is based on a retrospective analysis, using approved drugs from two versions of DrugBank^1^.

#### Datasets

The data used in this research is third-party data, created by DrugBank and which can be accessed via the website: www.drugbank.ca/releases. Our DDI prediction evaluation is based on a retrospective evaluation [8, 20], using approved drugs from two versions of the DrugBank database: 5.0.0 (from June 2016) and 5.1.1 (from July 2018). Major changes were made between the versions; specifically, a large number of interactions were added to the more recent version. For the validation process, we randomly split the training set based on version 5.0.0. 20% of randomly selected existing and non-existing interactions from the training set were used as the validation set. Versions 5.0.0 and 5.1.1 contain 1, 440 and 2, 149 approved drugs respectively. Here, we use only the intersecting set of drugs between the versions. The training set consists of 1, 036, 080 drug pairs, 45, 296 positive interactions and 990, 784 negative (potential) interactions. The test set consists of 1, 036, 080 drug pairs, 248, 146 positive interactions and 787, 934 negative (potential) interactions.

#### Baselines

**Fig. 2:**
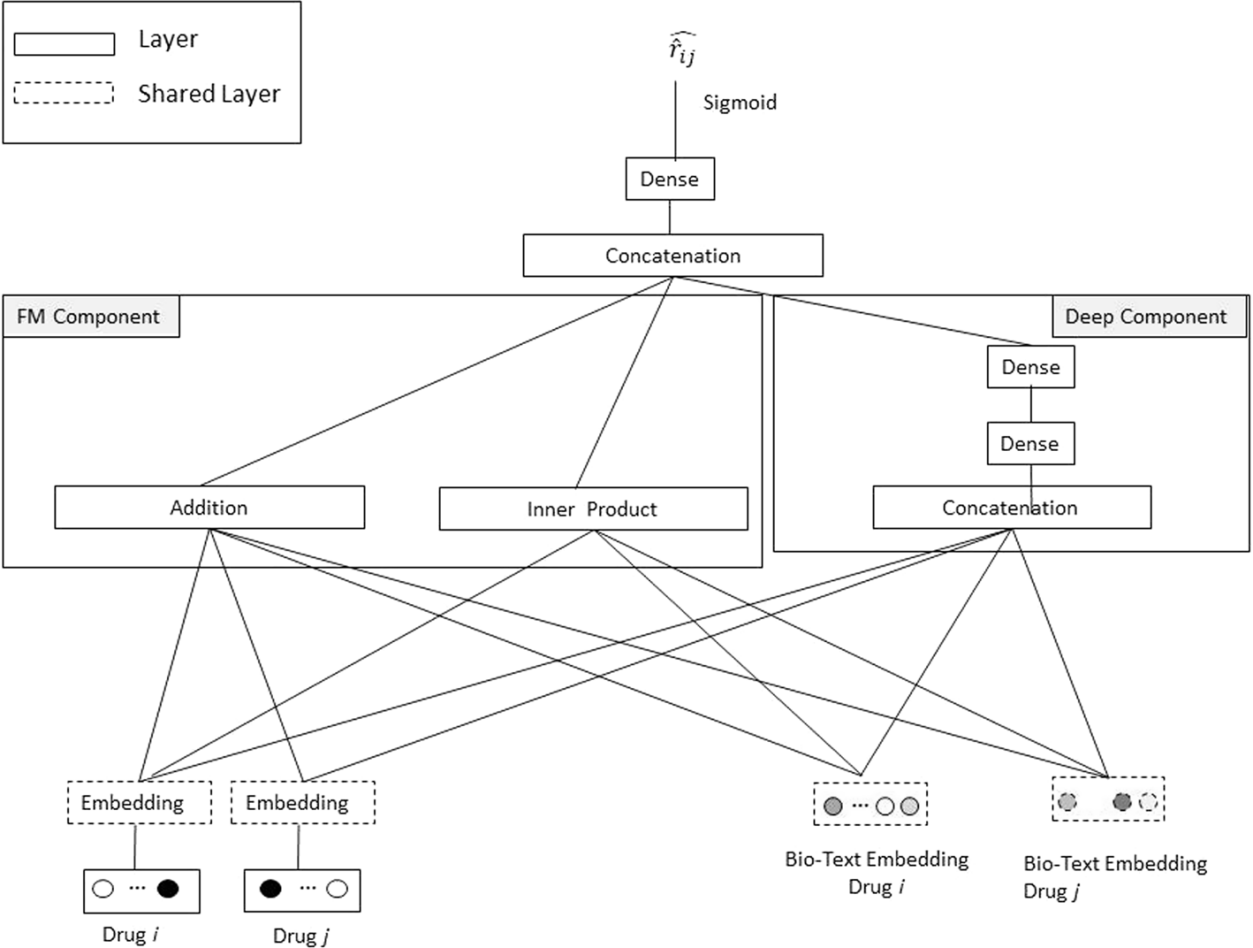
DeepFM architecture [21] for Drug–drug interaction prediction. The factorization machine (FM) and deep components share the same input raw feature vector, which enables DeepFM to learn low and high-order feature interactions simultaneously from the raw input features. The FM component learns first and second-order interactions using addition units and a number of inner product units. The deep component is a feed-forward neural network which learns higher-order interactions. The input layer includes the drugs’ pre-trained bio-text embedding and the drugs’ ids encoded as one-hot vectors. An embedding representation of the drugs is learnt as part of the overall learning architecture. The embedding layers are shared between the first and the second input drugs. The network output is the Drug–drug interaction score between the two input drugs

We compared our approach and the contribution of the Bio-Text embedding to DDI prediction to the following state-of-the-art approaches:

- *Adjacency matrix factorization (AMF)* [8] Matrix factorization on the adjacency matrix of Drug–drug interactions is applied. The latent factors for each drug are shared between rows and columns. Then AMF exploits adaptive moment estimation (Adam) optimization to optimize the weights of the element-wise multiplication and biases.
- *Adjacency matrix factorization with propagation (AMFP)* [8] An extension of the AMF approach which adds a step to propagate the latent factors of each drug to the interacting drugs.
- *Integrated similarity-constrained matrix factorization (ISCMF)* [9] An extension of the Matrix Factorization (MF) approach which constrains the latent factors by using an integrated similarity matrix. To allow a fair comparison with early stage available data, we adapted ISCMF to calculate the similarity of drugs based on the drugs’ concept embeddings. Additional methods were proposed based on the idea of constraining the latent factors by using an integrated similarity matrix [7]. Here, we used ISCMF as a baseline.
- *DeepFM* [21] This approach is considered the state-of-the-art model for combining an entity’s interactions with an entity’s characteristics. It consists of two components, an FM component and deep component that share the same input. The FM component is the factorization machines (FM) model, which models all interactions between variables using factorized parameters [22], enabling it to estimate interactions even in problems with high sparsity. It models pairwise feature interactions as an inner product of latent vectors between features. The deep neural network component is able to effectively learn higher-order feature interactions. We evaluated three flavors of the DeepFM approach. The model architecture is presented in Fig. 2.
  The drug ID and drug concept ID are considered two feature families. The drug ID embeddings are trained as part of the model, and for the concept ID embeddings, pretrained values are used. Since the sizes of the optimal drug ID and concept ID embeddings are different, we added an adaptation layer to the drug concept embedding which is used before calculating the interaction components of the factorization machine.
  *DeepFM (FM)* Only the FM component of the DeepFM model is used for prediction.
  *DeepFM (Deep):* Only the deep component of the DeepFM model is used for prediction.
- *Directed message passing neural network (Chemprop)* The authors of Chemprop [23], a message passing neural network for molecular property prediction, suggest processing the chemical structure graph with a message passing neural network (MPNN). Following Chemprop’s success in discovering a new antibiotic [24], we use chemprop for generating DDI predictions based on the molecules’ chemical structure by utilizing a graph convolutional model and refer to it as *Chemprop* in this paper. The MPNN framework consists of two phases: (1) the *message passing phase*, in which the molecule is represented by a latent representation; this phase runs in several iterations to update the bonds’ and atoms’ latent representation; (2) the *readout phase*, in which a readout function is used to compute the prediction using the representation of the whole graph. Using the MPNN framework can result in a noisy graph and a less accurate representation due to totters [25]. Therefore, the Chemprop framework employs a directed MPNN (D-MPNN) in which the messages are associated with directed edges (bonds) instead of the atoms. The Chemprop baseline we use in this research takes two molecules as input, each molecule is processed by a single Chemprop model, the weights of the two models are shared. Finally, a prediction is obtained by concatenating the molecules’ representation created in the readout phase, applying a feed-froward neural network and a binary cross-entropy loss.
- *CASTER* The CASTER [26] framework uses functional representations to represent the different drugs, i.e., the authors used the most frequent substructures shared by a pair of drugs. Then, the authors used an unsupervised encoder-decoder network to create a latent representation for each drug’s functional representation.
  The authors also represented the most frequent SMILES substructures with a designated latent representation. Latent vectors of the functional representation are mapped to the same latent space of the SMILES substructures. Linear coefficients are used as features to predict the DDIs. In the training phase, the authors minimized two loss functions: (1) reconstruction loss, to represent the drugs’ latent functional representation, and (2) prediction loss, which is a binary cross-entropy loss function, to provide the prediction of DDIs.
- *SSI-DDI* [27] is a recently released DDI prediction system that extracts features straight from raw molecular graph representations of pharmaceuticals. The model is based mainly on several graph attention (GAT) layers followed by a co-attention layer. SSI-DDI is trained to distinguish between different drug interaction types; the algorithm samples negative instances from the training set. Here, to adapt SSI-DDI to the current problem, we train SSI-DDI on just a single target attribute, because the current work defines the task as binary.

#### Metrics

The primary evaluation metric used is the area under the ROC curve (AUROC). We also assess the area under the precision-recall curve (AUPR), because it is more reliable in the case of an unbalanced dataset, as in our case. Lastly, we plot precision@n and recall@n which evaluate the top n most confident predictions of the model. This metric is important, since our machine learning model will determine which drug interactions will be tested in a lab. We acknowledge the importance of precision over recall in the DDI problem, and therefore we plot the two precision graphs in addition to the other metrics.

#### ABTE evaluation

We evaluated the following flavors of the ABTE approach:

- *Text concate* The bio-text embedding vector of the two drugs are concatenated and fed to a multilayer feed forward neural network classifier.
- *Text multiply* The bio-text embedding vectors of the two drugs are multiplied (using element-wise multiplication), concatenated with a bias component, and fed to a multilayer neural network classifier.
- *Text concate, feature combination* In this model, the drug ID embedding and the drug bio-text embedding are integrated as part of a single neural network which predicts the DDI interaction. The text embedding vectors of the two drugs and the element-wise multiplication of the drug ID embedding vectors are concatenated. The concatenated layer is then fed to a multilayer neural network classifier.
- *Text multiply, feature combination* The drug ID embedding and drug bio-text embedding are integrated as part of a single neural network which predicts the DDI integration. The text embedding vectors of the two drugs and the drug ID embedding are multiplied separately. The element-wise multiplication of each component is then concatenated and fed to a multilayer neural network classifier.
- *Text concate, stacking* The prediction of the bio-text concate model and the AMFP model are fed to a stacking model (multilayer neural network) to provide the final DDI interaction prediction.
- *Text multiply, stacking* The prediction of the bio-text multiply model and the AMFP model are fed to a stacking model (multilayer neural network) to provide the final DDI interaction prediction.

Table 1 presents a comparison between the different ABTE flaovrs, while incorporating text embeddings based on different models for the DDI prediction task. As can be seen, the “Text Concate with Stacking” flavor, an ensemble that combines the predictions based on the drugs’ text embedding with the AMFP predictions is superior for the DDI prediction task. A medical NER stage for aggregating different wording for the same drug is superior to the other drug text embedding models. In this use case, the DDI classification using the BioBERT text embedding approach provides lower accuracy since the text embedding includes only the drug name or concept, and there is no sentence context.

Table 2 presents the same evaluation results presented in Table 1, but in this case, we evaluate only the interactions of the rare drugs. Tuning ABTE’s parameter *M*, resulted in an optimal value of three. Hence, here, we considered new drugs as drugs with less than three interactions in the training dataset. In this scenario, the best-performing model is the “Text Concate” which is based only on the text embedding.

Table 3 presents a comparison of the ABTE best-performing approaches with state of the art Matrix Factorization extensions. The comparison includes state of the art models for DDI prediction, as well as hybrid approaches to combine text embedding and known interactions.

As can be seen, when evaluating the complete test set, the AMFP approach is superior to the standalone approach based only on the drugs’ text embedding. However, a hybrid approach using an external combiner provides a slight improvement in the prediction classification metrics. The DeepFM approach’s prediction accuracy is similar to the AMF approach. This is because the drug ID interactions’ contribution to the prediction score is significantly higher than the contribution of the text embedding interactions and the standalone features.

In the case of rare drugs that have a low number of known interactions during the model training period, the performance of the “Text Concate” model which is based on text-embedding only is superior to that of the AMFP model and to the “Text Concate with Stacking” hybrid model.

In an analogy to recommender systems [28] where the interaction between drugs represents interactions between users and items, and the text embedding represents the item’s characteristics. A model based only on drug name text embedding is analogous to a content-based approach that leverages the item characteristics and is suitable for handling new items (i.e. the cold-start scenario). The AMFP, which is based on a matrix factorization approach, represents the neighbourhood approach where the similarity in interactions between users or items is leveraged to predict unknown interactions. In the case of rare drugs, the ISCMF approach which constrains the MF latent factors by using an integrated similarity matrix based on the drugs’ text embedding is superior to the AMFP approach which is based on known interactions only but has lower accuracy than the “Text Concate” approach. This comparison demonstrates the contribution of Bio-Text embedding to the DDI prediction task and the superiority of the ABTE hybrid approach with switching criteria compared to other MF extensions.

As part of our comprehensive evaluation, we compare the proposed approaches with existing works for DDI prediction, which are based on the drugs’ chemical structure. Table 4 presents the results of this comparison. On the complete dataset, the “Text Concate with Stacking” hybrid approach, achieves the best performance in terms of AUC and AUPR, followed by the AMFP model. The “Text Concate” model achieves the best performance on the rare drugs test subset. The “Text Concate” model is identical to the ABTE by the definition of ABTE’s switching criteria on rare drugs. Note that unlike the previous tables presented above, this comparison includes only small molecule drugs to allow a fair comparison with Chemprop, CASTER, and SSI DDI, which are based on the chemical structure of the drug and support only small molecule drugs. Unlike the “Text Concate”, “Text Concate with Stacking”, ISCMF and AMFP, which also support biological drugs. This comparison further demonstrates the usefulness of Bio-Text embedding to DDI prediction, especially in the case of rare drugs.

**Fig. 3:**
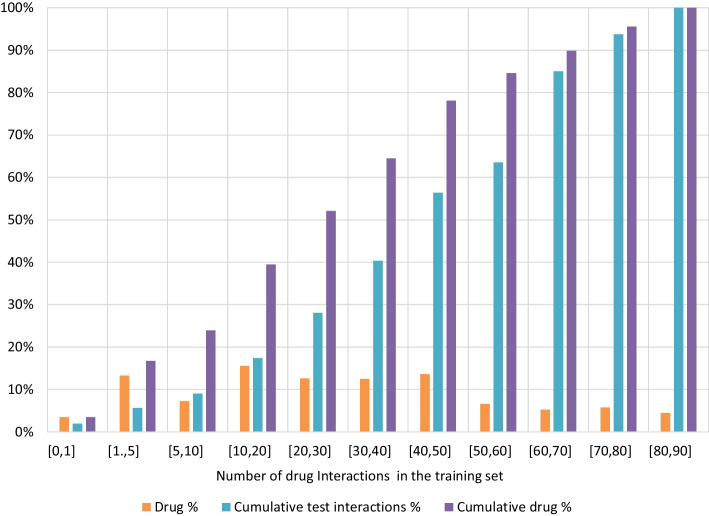
Drug distribution based on the number of interactions in the training set. The drugs % is the percent of drugs for the specified bin, the drug cumulative % represents the percent of drugs with a lower or equal number of the specified training interaction, and the cumulative test interactions % represents the percent of interactions in the test set for drugs with a lower or equal number of the specified training interaction bin

**Fig. 4:**
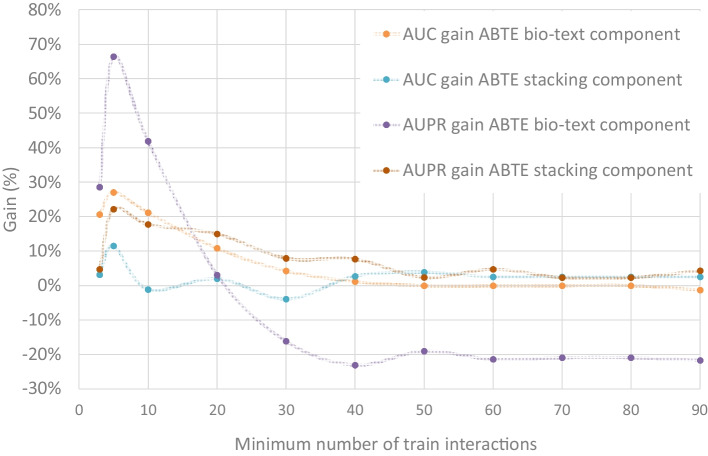
Gain of the ABTE text and stacking components compared to AMFP as a function of the number of drug interactions in the training set. Zero percent is AMFP’s performances

Figure 3 presents the distribution of drugs based on the number of interactions they have in the training set. It can be seen that about 25% of the drugs have less than 10 interactions in the training set. These drugs are responsible for 10% of the interactions in the test set. This set of drugs is of special interest, because it contains mainly drugs in the early stages of the drug lifecycle. To examine the ABTE approach superiority comparing to the AMFP approach, and to set its switching criteria, we evaluated the ROC AUC and AUPR AUC gain of the ABTE bio-text and stacking components comparing to the AMFP approach as a function of the number of interactions the drug has in the training set. The ABTE bio-text component uses the text concate approach, and the stacking component uses the text concate with external combiner approach. The results are presented in Fig. 4. As can be seen, when the number of interactions in the training set is lower or equal to 10 (about 20% of the drugs in the test set), the ABTE bio-text component (Text Concate) is superior to both the AMFP and the ABTE Stacking component. When the number of interactions in the training set is higher than 10, the ABTE Stacking provides a higher AUPR AUC, and its gain compared to the AMFP decreases as we add drugs with a higher number of interactions in the training set.

**Fig. 5:**
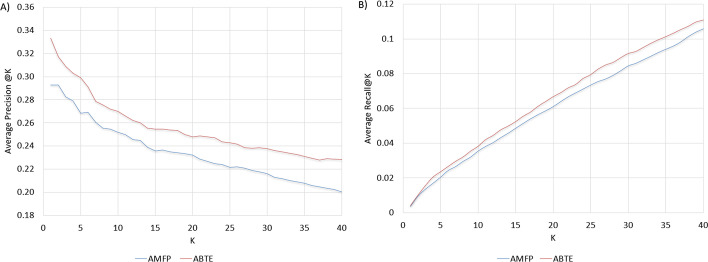
**a** Average precision of rare drug interaction prediction. The minimum number of drug interactions in the training set = 3. **b** Average precision of rare drug interaction prediction. The minimum number of drug interactions in the training set = 3

In Fig. 5a, we present the average precision@K for all of the rare drugs that have less than three interactions in the training set, and in Fig. 5b, we present the average recall@K for the interactions of the same drugs. As can be seen, the ABTE approach is superior to the AMFP approach for both precision@K and the recall@K metrics. As the machine learning model predictions will determine which drug interactions will be tested in the lab, higher precision@K means that fewer experiments will be required to detect possible interactions for new drugs.

### Pregnancy drug safety classification

**Table 5:** Pregnancy drug safety classification

Model	Modalities	Text embedding	AUC	AUPR
XGBoost	DrugBank category	–	0.87	0.86
XGBoost	Molecular structure	–	0.76	0.72
XGBoost	Text	Concept embedding	0.88	0.85
XGBoost	Text	Word embedding	0.77	0.74
XGBoost	Text	BioBERT embedding	0.74	0.70
XGBoost	Text and molecular structure	Concept embedding	0.88	0.84
ExtraTrees	DrugBank category	–	0.88	0.86
ExtraTrees	Molecular structure	–	0.78	0.72
ExtraTrees	Text	Concept embedding	0.87	0.84
ExtraTrees	Text	Word embedding	0.79	0.76
ExtraTrees	Text	BioBERT embedding	0.77	0.74
ExtraTrees	Text and molecular	Concept embedding	0.88	0.85

To further evaluate the contribution of text embedding to medical prediction tasks related to new drugs, we compared the accuracy of different machine learning models to predict pregnancy drug safety based on different related modalities. The modalities we evaluated are the different biomedical text embeddings, the drug’s molecular structure [29], and the DrugBank drug category. DrugBank’s drug category combines the drug’s ATC codes of different levels, and other manually curated drug information [30]. The DrugBank category is considered the most informative modality for drug safety classification, but it is not available during the early stages of a drug’s development.

In this experiment, a dataset of 124 drugs, labeled manually by a domain expert, was used to evaluate the accuracy of two prediction models: ExtraTrees and XGBoost. The AUC and AUPR metrics, using 10-fold cross-validation were used for the comparison. The code published by [30] for feature engineering and model generation was used.

Table 5 presents the evaluation results. It can be seen that similarly to the DDI prediction task, using the concept embedding modality was superior to the other two Bio-Text embedding modalities, as well as the use of the drug’s molecular structure modality. A multimodal model which combines the drug’s molecular structure and Bio-Text embedding information, which is available during the early stages of the drug’s approval process, is comparable to a model based on the DrugBank category in terms of the AUC.

DrugBank’s categories contain more than 2,000 binary features, crafting these features requires manual, labour-intensive categorization and a solid scientific literature focused on each drug. The results on Table 5 suggest that unstructured features can be used for classification of drugs, eliminating the need to manually categorize drugs. The results suggest that accurate drug-related predictions are feasible on numerous drugs, using Bio-Text embeddings created automatically from the literature and not only on thousands of approved compounds using manually categorized drugs.

## Conclusion

In this research we assert, that Bio-Text drug embedding can improve the performance of machine learning models for predicting drugs’ characteristics. Our findings indicate that Text Bio-Concept embedding, which incorporates biomedical knowledge into the embedding process, outperforms biomedical corpora word embedding and embeddings originating from BioBERT. We support our claim by demonstrating the superiority of a hybrid model which uses Concept Bio-Text embedding and historical interactions to predict DDIs, as well as by evaluating the contribution of Bio-Text embedding to machine learning classifiers used to predict the safety of drugs during pregnancy. These results demonstrate that Bio-medical concept embeddings provide valuable information that can be used to predict drug’s characteristics. It is more valuable for relatively new drugs where the amount of other source of information is limited. However, relying on Bio-medical concept embeddings limits the explainablity of the machine learning model, because Bio-medical concept embeddings are not generally understandable to humans.

We introduce ABTE, a hybrid approach which combines drug’s Bio-Text embeddings with drug’s known interactions and demonstrate its superiority over state-of-the-art approaches for DDI prediction. We show that the improvement is more significant for new drugs with a small amount of known interactions, while a subtle improvement is demonstrated for known drugs with a fair amount of known interactions.

In future research, we would like to evaluate the contribution of using bio-text embedding to improve the accuracy of additional drug-related prediction tasks such as drug re-purposing, drug-target interaction, and drug-disease effectiveness. In addition, we would like to further enhance the ABTE hybrid approach for medical interaction predictions tasks by combining the matrix factorization approach with graph neural networks to better capture the nonlinear associations between different biomedical entities.

## Data Availability

The datasets generated and/or analysed during the current study are available in the DrugBank repository. The model scripts are available at https://github.com/asnatm/ddi.git. Text embeddings are available at PubTator’s central chemical mapping.

## Footnotes

1 Third-party data owned by The Governors of the University of Alberta and managed by OMx Personal Health Analytics Inc. The dataset can be accessed via the website: www.drugbank.ca/releases
